# A cell-free system for functional studies of small membrane proteins

**DOI:** 10.1016/j.jbc.2024.107850

**Published:** 2024-10-01

**Authors:** Shan Jiang, Gülce Çelen, Timo Glatter, Henrike Niederholtmeyer, Jing Yuan

**Affiliations:** 1 Max Planck Institute for Terrestrial Microbiology and Center for Synthetic Microbiology, Marburg, Germany; 2 Technical University of Munich, Campus Straubing for Biotechnology and Sustainability, Straubing, Germany

**Keywords:** cell-free synthesis, small protein, membrane protein, lipid sponge droplet, protein function discovery

## Abstract

Numerous small proteins have been discovered across all domains of life, among which many are hydrophobic and predicted to localize to the cell membrane. Based on a few that are well-studied, small membrane proteins are regulators involved in various biological processes, such as cell signaling, nutrient transport, drug resistance, and stress response. However, the function of most identified small membrane proteins remains elusive. Their small size and hydrophobicity make protein production challenging, hindering function discovery. Here, we combined a cell-free system with lipid sponge droplets and synthesized small membrane proteins *in vitro*. Lipid sponge droplets contain a dense network of lipid bilayers, which accommodates and extracts newly synthesized small membrane proteins from the aqueous surroundings. Using small bacterial membrane proteins MgrB, SafA, and AcrZ as proof of principle, we showed that the *in vitro*–produced membrane proteins were functionally active, for example, modulating the activity of their target kinase as expected. The cell-free system produced small membrane proteins, including one from human, up to micromolar concentrations, indicating its high level of versatility and productivity. Furthermore, AcrZ produced in this system was used successfully for *in vitro* co-immunoprecipitations to identify interaction partners. This work presents a robust alternative approach for producing small membrane proteins, which opens a door to their function discovery in different domains of life.

Small proteins also named mini-proteins (1), micro-proteins (2), or small peptides (3) were overlooked in the past due to their small size (less than 50 amino acids in prokaryotes and less than 100 amino acids in eukaryotes) (3, 4, 5). Small proteins are directly translated from small ORFs (sORFs) that often start with noncanonical start codons, which increases the difficulty of differentiating valid sORFs from noncoding RNAs (3, 4). With the advances in bioinformatics and the application of ribosomal profiling, hundreds to thousands of sORFs have been identified in recent years (6, 7, 8). While some have low stability and have no apparent contribution to cell fitness in the conditions tested (9), many are conserved, predicted to have a putative transmembrane helix, and may be involved in cell signaling and communication (6, 10, 11).

Small membrane proteins constitute a significant portion of newly discovered small proteins. They are found to be involved in diverse biological processes across domains of life, ranging from bacterial virulence regulation to immunosurveillance in mammalian cells (reviewed in (12, 13)). Well-characterized bacterial small membrane proteins primarily act as regulators by integrating into large protein complexes, such as KdpF facilitating potassium transport complex formation (14), AcrZ specifying substrates for the AcrBA-TolC drug efflux pump (15), and Prli42 anchoring the stressosome to the bacterial membrane (16). Small membrane proteins also target individual larger membrane proteins, regulating their stability and function. For example, MgrB and SafA participate in cell signaling by regulating the activity of the sensor kinase PhoQ (17, 18, 19, 20). MgtS and MntS regulate magnesium and manganese transport by targeting the respective transporter proteins (21, 22). Lastly, some small membrane proteins directly target the cell membrane, acting as a detector for membrane curvature (23) or as type I toxins regulating membrane permeability (24).

Despite the knowledge and experience gained from the well-characterized small proteins, the function of most newly discovered small membrane proteins awaits further investigation. In contrast to soluble proteins, small membrane proteins are challenging to produce *via* chemical synthesis due to their hydrophobicity. Overexpression of recombinant small membrane proteins *in vivo* may induce general stress or even be toxic to cells (11, 25, 26, 27). These difficulties become major roadblocks for functional studies.

Cell-free protein synthesis (CFPS) has been used for membrane protein production *in vitro* to avoid potential cytotoxicity and protein aggregation, to improve yield, and to characterize membrane protein functions (28, 29, 30, 31). Commonly used hydrophobic supplements in a cell-free system for membrane protein production include detergents, nanodiscs, and liposomes, which prevent precipitation and support the synthesis and folding of membrane proteins during synthesis. Liposomes closely mimic cellular membranes. Their membrane surface area correlates with the yield of folded and active membrane proteins (32, 33), underscoring the importance of a suitable membrane environment for functional membrane protein production in CFPS. Liposomes also generate separate compartments, which are required for the functional assessment of channel and transporter proteins. However, the main drawback of liposomes is their instability in time and response to osmolarity changes. Nanodiscs (34) are a more stable alternative to liposomes, but both require specialized equipment and multiple steps for their preparation. In addition, both systems are limited in the membrane surface area they can provide.

Unlike liposomes and nanodiscs, lipid sponge droplets contain dense non-lamellar lipid bilayer networks, providing an extensive membrane surface for membrane protein insertion. They have a high water content, harbor nanometric aqueous channels, and thus simultaneously accommodate hydrophilic components of a cell-free system. Lipid sponge droplets are simple to prepare and generated *via* spontaneous assembly of the single-chain galactolipid N-oleoyl β-D-galactopyranosylamine (GOA) and non-ionic detergents such as octylphenoxypolyethoxyethanol (IGEPAL). In aqueous solutions, the two amphiphiles form micrometer-sized coacervate-like droplets that are stable in a wide range of conditions (35, 36). Despite being a nonbiological lipid, the thickness of GOA membranes (35.25 Å) (37) is comparable to that of a biological membrane (37–40 Å) (38). While two transmembrane proteins, diacylglycerol kinase and cytochrome c oxidase, were enzymatically active when reconstituted in lipid sponge droplets (35), the insertion or activity of small membrane proteins in lipid sponge droplets has not been tested.

In this study, we combined lipid sponge droplets with a minimal, defined cell-free system (PURE system) (39) and produced small membrane proteins *in vitro*. The PURE system contains all necessary *Escherichia coli* translation factors, ribosomes, and the T7 RNA polymerase in purified form. Compared to CFPS systems based on cellular lysates, the advantage of the PURE system is that its components are known and limited to a minimal set. We chose the PURE system because of its compatibility with the droplets and to avoid potential interference with downstream functional studies of the synthesized small membrane proteins. Using *E. coli* proteins MgrB and SafA as proof of principle, we showed that the small proteins were successfully synthesized and localized in the lipid sponge droplets. The yield was sufficient for downstream functional assays, and the lipid sponge was required to reach this level of protein production. The synthesized MgrB and SafA were functionally active, modifying the kinase activity of their target protein PhoQ *in vitro*. Successful synthesis of two other small membrane proteins AcrZ from *E. coli* and sarcolipin from human demonstrates the versatility of this cell-free system, and we present strategies to increase the yield of the synthesized small membrane proteins to micromolar concentrations. Lastly, we successfully co-immunoprecipitated the interacting proteins of AcrZ from solubilized cell membranes, suggesting that this cell-free system is suitable for target discovery of small membrane proteins forming stable complexes with their targets.

## Results

### Cell-free biosynthesis of small membrane proteins MgrB and SafA

**Figure 1:**
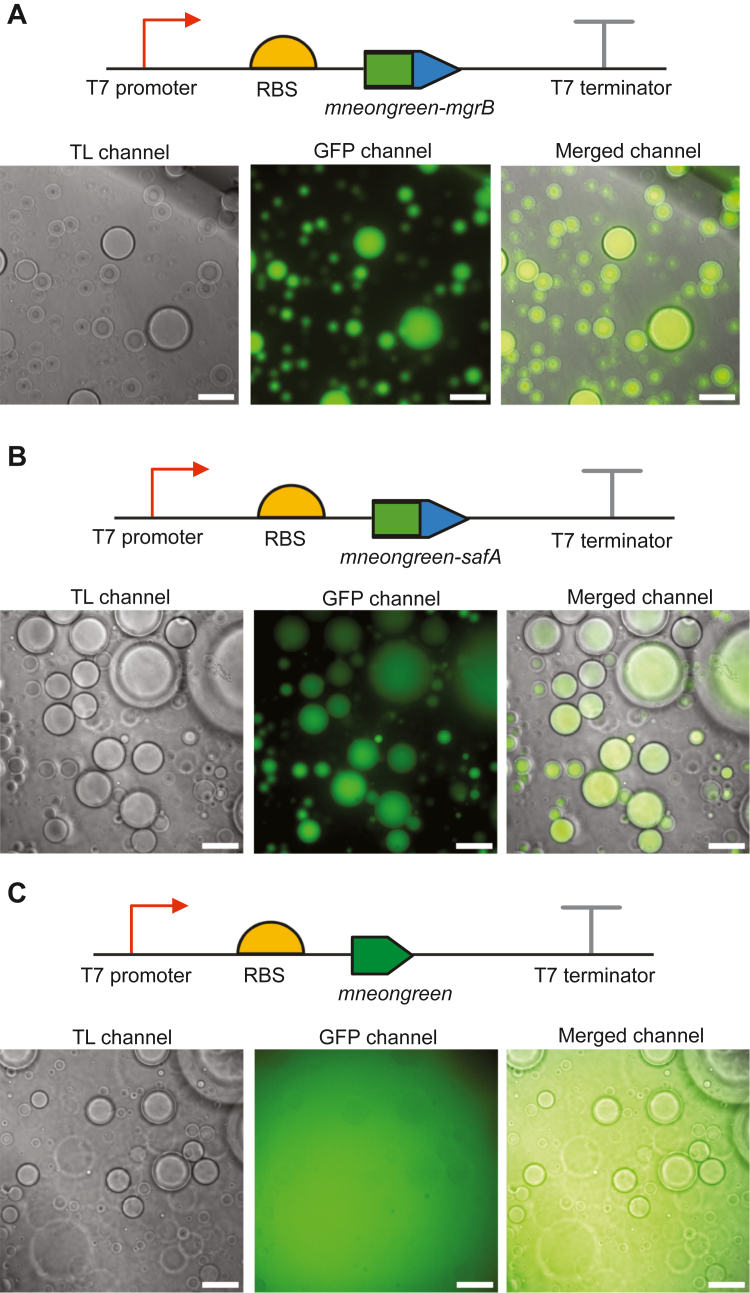
**Cell-free synthesis of small membrane proteins in the presence of lipid sponge droplet**. The mNeonGreen labeled MgrB (*A*), SafA (*B*), and mNeonGreen control (*C*) were synthesized in the cell-free system in the presence of lipid droplets. Representative images from the transmitted light (TL), GFP fluorescence, and merged channels are shown with the corresponding DNA template scheme above (Scale bar represents 50 μm). Data are representative of at least three independent experiments.

**Figure 2:**
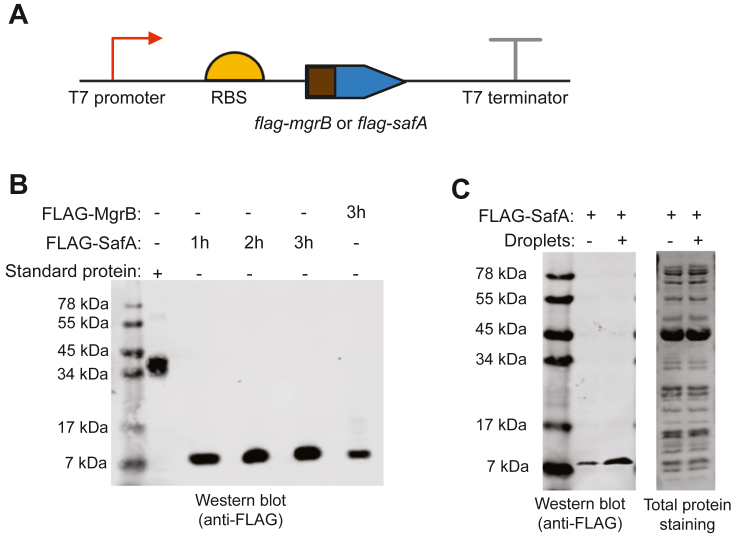
**Quantification of synthesized small membrane proteins in the cell-free system**. *A*, design of the linear DNA templates. *B*, Western blot analysis of the synthesized MgrB and SafA in a 5 μl reaction. A FLAG-tagged standard protein (0.1 μg) was used as a positive control. *C*, cell-free synthesis of SafA with or without lipid sponge droplets. Data are representative of three independent experiments.

We started with two well-studied *E. coli* proteins MgrB (47aa) and SafA (65aa) to test the cell-free system for small membrane protein production. DNA templates containing T7 promoter and the ORFs of MgrB and SafA were constructed (Fig. 1) and used to initiate protein synthesis in the cell-free system with lipid sponge droplets. To visualize CFPS, we added an N-terminal mNeonGreen tag to the small membrane proteins. Additionally, a DNA template for synthesizing only mNeonGreen was used as a control. We observed a gradual increase in green fluorescence during incubation, which reached a plateau after around 3 h (Fig. 1 and Movie S1). For the synthesis of mNeongreen-MgrB and mNeongreen-SafA, the fluorescence primarily localized to lipid sponge droplets (Fig. 1 and Movie S1). Conversely, the mNeongreen control sample showed diffuse fluorescence in the field of view with lipid sponge droplets having a slightly lower fluorescent intensity (Fig. 1*C*). These results suggested that the mNeongreen-tagged small membrane proteins were produced in the cell-free system and that their hydrophobicity led to sequestration of the fusion proteins into the membrane-rich droplet phase. To quantify MgrB and SafA synthesized in the cell-free system, we separately introduced a FLAG epitope tag to the N terminus of the proteins (Fig. 2*A*). Using a commercially available FLAG-tagged protein as a reference in Western blot analysis, we quantified that approximately 600 nM FLAG-SafA was produced in the cell-free system after 3 hours and about 300 nM for FLAG-MgrB (Figs. 2*B* and S1). This concentration is comparable (in terms of mg/ml) to the reported concentrations reached for larger membrane proteins in PURE reactions supplemented with liposomes (40).

Small membrane proteins, such as MgrB and SafA, typically contain a single transmembrane helix (Table S1). To determine whether lipid sponge droplets were required for such bitopic integral membrane protein synthesis *in vitro*, we produced and compared FLAG-SafA with the PURE system in the presence and absence of lipid sponge droplets. The Western blot results showed that a higher amount (2.73 times) of SafA was produced in the presence of lipid sponge droplets (Figs. 2*C* and S2), indicating that the hydrophobic environment is beneficial despite the seemingly simple tertiary structure of SafA. The lipid sponge droplets facilitated the reconstitution of the synthesized protein, likely prevented protein aggregation, and thus increased the yield of SafA during CFPS. Along similar lines, previous work on CFPS of membrane channel proteins showed that the production of folded and active protein correlated with the amount of supplemented liposomes (32, 33). While the membrane surface area liposomes can offer is limited, lipid sponge droplets are filled with a dense membrane environment and can be added to CFPS reactions at high concentrations (35).

### Functional analysis of the synthesized MgrB and SafA

**Figure 3:**
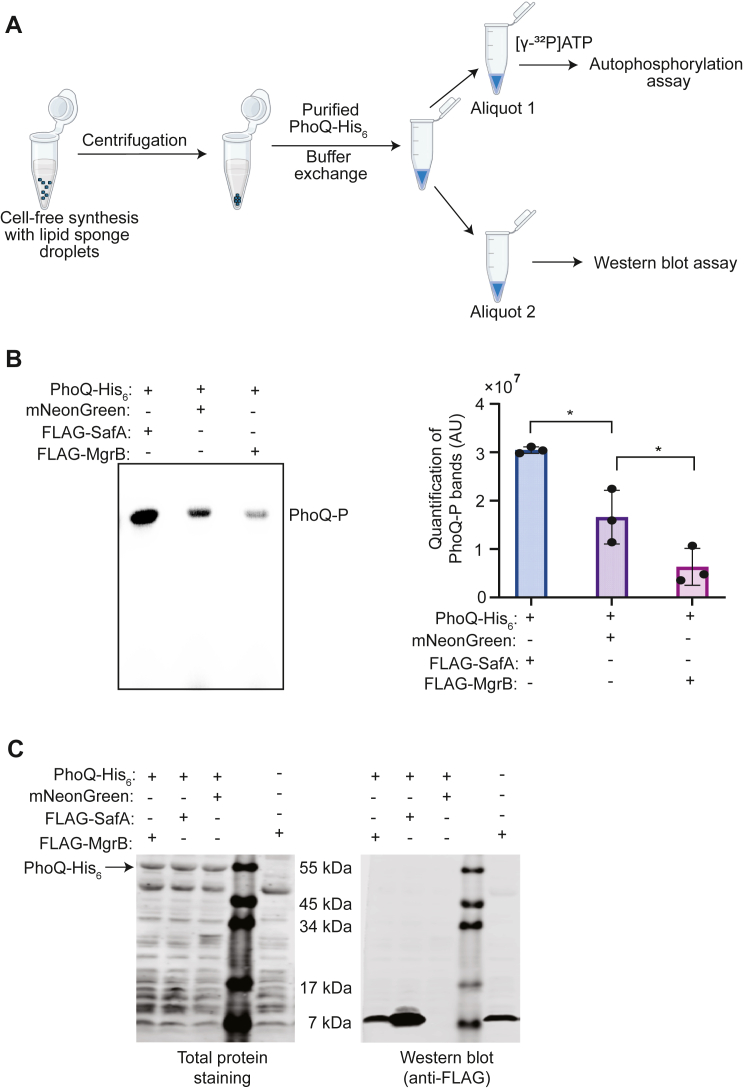
**Functional assay of synthesized small membrane proteins MgrB and SafA**. *A*, scheme of the functional assay. *B*, autophosphorylation of PhoQ in the presence of synthesized proteins. The reactions were carried out at room temperature for 30 min. The phosphorylated PhoQ was separated from free [γ-^32^P]ATPs by SDS-PAGE and detected with a phosphorimager (*left*). The bands were quantified using ImageJ software (*right*). Error bars represent the SDs from three independent experiments. *C*, the total protein and Western blot analysis of the reaction mix in the functional assay. The unlabeled lanes in (*C*) are the molecular weight markers. Data are representative of at least three independent experiments.

The function of MgrB and SafA can be tested by following the activity of their target protein PhoQ. The sensor kinase PhoQ catalyzes an autophosphorylation reaction, where it utilizes ATP to phosphorylate a conserved histidine residue. MgrB and SafA directly interact with PhoQ. MgrB inhibits the autophosphorylation of PhoQ, while SafA is an activator. To test the regulatory function of the synthesized small proteins, we performed PhoQ autophosphorylation assays with mNeonGreen as a negative control (the experimental scheme shown in Figure 3*A*). To introduce the synthesized FLAG-SafA and FLAG-MgrB to the reaction, we spun down the droplets after CFPS and replaced the supernatant with purified recombinant PhoQ protein in phosphorylation buffer. Centrifugation allowed us to enrich lipid sponge droplets and the small membrane proteins localized within; however, some residual aqueous components of the cell-free system were carried over to the autophosphorylation reaction. Lauryl maltose neopentyl glycol (LMNG) detergent in the PhoQ sample then dissolved lipid sponge droplets under these conditions, allowing interactions to occur. The reaction mix was divided into two aliquots. One proceeded to autophosphorylation assays by adding radioactively labeled [γ-^32^P]ATP. The other was used for SDS-PAGE/Western blot analysis to verify the production of the small proteins and the amount of PhoQ. It is noteworthy that we used an excess amount of PhoQ in the reactions to ensure detectable signals since the carried-over CFPS components inhibited PhoQ function. We observed an increase in radioactively labeled PhoQ in the presence of the synthesized FLAG-SafA and a decrease in autophosphorylation with the synthesized FLAG-MgrB compared to the mNeonGreen negative control (Fig. 3, *B* and *C*), indicating that the synthesized small membrane proteins are functionally active.

With the same setup, we synthesized and tested the function of MgrB and SafA without epitope tags and observed similar results with autophosphorylation assays (Fig. S3). Altogether, the results suggested that our cell-free system can produce functional small membrane proteins in sufficient amounts for downstream analysis *in vitro*. The ability to synthesize native functional small membrane proteins is especially advantageous for studying the ones sensitive to epitope or affinity tags.

### Cell-free synthesis of other small membrane proteins

**Figure 4:**
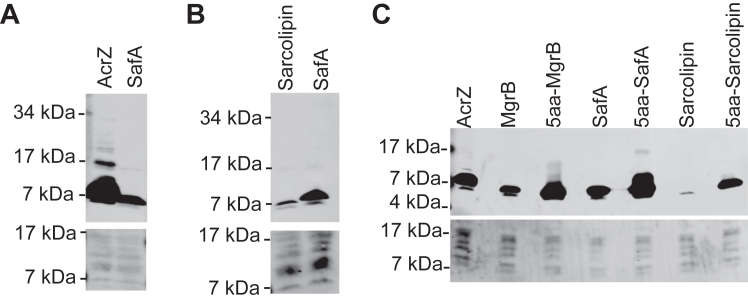
**Cell-free synthesis of AcrZ, sarcolipin, and yield optimization**. Western blot analysis of the synthesized C-terminal FLAG-tagged AcrZ from *Escherichia coli* (*A*) and N-terminal FLAG-tagged sarcolipin from human (*B*) are shown with the synthesis of SafA as a positive control. *C*, Western blot analysis of synthesized small membrane proteins with or without N-terminal 5-amino acid (5aa) insertions. The corresponding total protein stain of the PVDF membranes serves as loading control. Data are representative of three independent experiments. Based on the results in Figures 2 and S1, about 600 nM FLAG-SafA was produced in a 5 μl reaction. The yield of the other small proteins synthesized with the same reaction volume was estimated using FLAG-SafA as a reference in Fig. S7.

To test the versatility of the cell-free system, we applied it to synthesize two other small membrane proteins, AcrZ from *E.coli* and sarcolipin from human. AcrZ is an integral membrane protein. It interacts with the drug efflux protein AcrB and forms a 3:3 hexameric membrane protein complex, which can bind to AcrA and TolC, forming a complete drug efflux pump (15, 41). Functionally, AcrZ regulates the substrate specificity of the drug efflux transporter (15, 41). Sarcolipin is an essential regulator of the sarco-/endoplasmic reticulum Ca^2+^-ATPase in human myocytes (42). It prevents the pumping of calcium cations out of the cytosol without inhibiting the ATPase activity, thus converting the chemical energy stored in ATP to heat (42). After codon optimization, we constructed the DNA templates and performed CFPS of AcrZ and sarcolipin in the presence of lipid sponge droplets. A FLAG-tag was introduced for quantification *via* Western blot analysis. With SafA as a positive control, we observed that AcrZ and sarcolipin were produced using the cell-free system. The yield of AcrZ was about 2.4 times more than SafA, reaching the micromolar range (about 1.4 μM) (Figs. 4*A* and S4). Human sarcolipin was synthesized less (about 90 nM). One reason for the lower yield could be the polar residue (Asn11) in its transmembrane helix (Table S1, Figs. 4*B*, and S5) hindering protein partitioning to lipid sponge droplets. Indeed, an N to L substitution increased the yield (approximately 2.4 times) of sarcolipin (Fig. S6).

A previous study showed that the mRNA sequence immediately downstream of the start codon affects translation efficiency (43). To increase the yield of MgrB, SafA, and sarcolipin to a level comparable to that of AcrZ, we then copied the DNA sequence coding for the first five amino acids of AcrZ and inserted it between the start codon and the N-terminal FLAG tag in the corresponding ORFs. When using these modified DNA templates for cell-free synthesis, we observed a drastic increase in protein yield (Fig. 4*C*). All three small membrane proteins were produced near or at micromolar concentrations (Fig. S7), significantly more than the previously reported concentrations for membrane protein synthesis in PURE reactions with liposomes (40). Taken together, our results demonstrate the versatility and productivity of the cell-free system in generating small membrane proteins.

### Target discovery using the synthesized small membrane proteins

**Figure 5:**
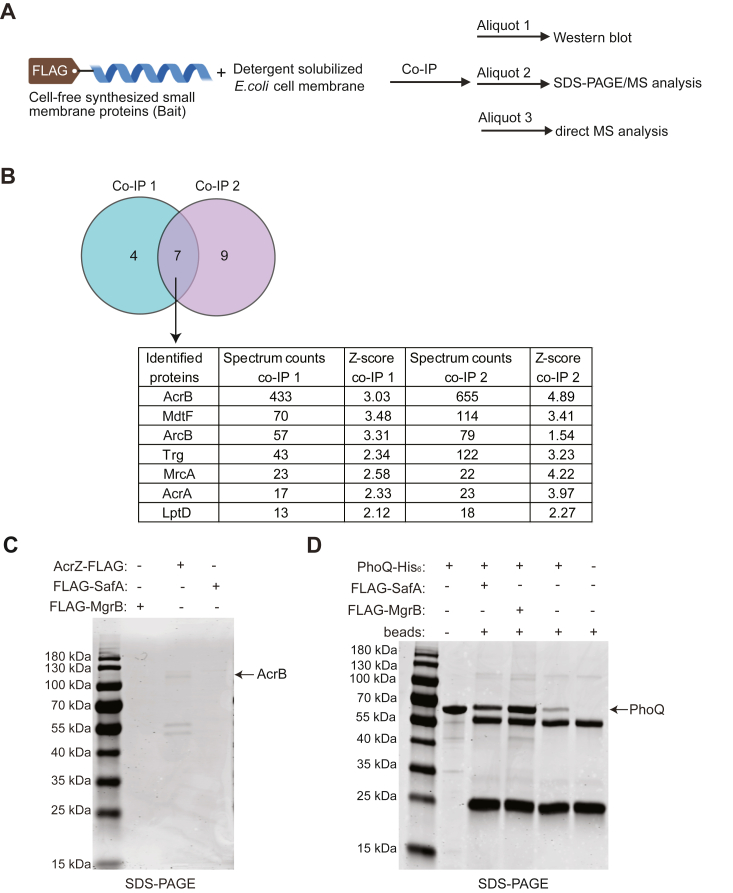
**Identification of interacting targets of small membrane proteins.***A*, scheme of the *in vitro* Co-IP assay. *B*, using synthesized AcrZ-Flag as bait, co-immunoprecipitated proteins were identified *via* MS. Seven proteins with a minimum Z-score of 2 in two independent experiments are shown and considered enriched (see Experimental procedures for details of Z-score calculation). The spectrum counts for each enriched protein are listed. *C*, SDS-PAGE analysis of eluates from co-IPs using FLAG-tagged MgrB, AcrZ, and SafA as baits. *D*, SDS-PAGE analysis of eluates from co-IPs using purified recombinant PhoQ instead of detergent-solubilized cell membrane to interact with the indicated FLAG-tagged bait proteins. The SDS-PAGE is a representative of three independent experiments.

To determine whether the *in vitro* synthesized small membrane proteins were suitable for target discovery, we used MgrB, SafA, and AcrZ fused to a FLAG tag as bait to identify their interaction partners *via* co-immunoprecipitation (co-IP) (Fig. 5*A*). Due to their short length, small proteins are more prone to function interference by affinity tags in general. To ensure their functionality, we tagged SafA, MgrB, and AcrZ based on previous studies (15, 17, 44). The tagged small membrane proteins were synthesized using the cell-free system as described above. Meanwhile, we isolated the total membrane fraction from the corresponding *E. coli* KO strains (*ΔmgrB*, *ΔsafA*, and *ΔacrZ*). The membrane was solubilized with a detergent (LMNG)-containing buffer and incubated with the synthesized FLAG-tagged small membrane proteins. LMNG dissolved most lipid sponge droplets, allowing interactions between the synthesized small protein and components in the cell membrane. With anti-FLAG magnetic beads, proteins bound to bait were isolated and analyzed with mass spectrometry (MS), and the results showed that seven proteins were reproducibly enriched in the AcrZ co-IP samples with Z-scores above 2 (Fig. 5*B*, Files S1, and S2). AcrB, the known interactor of AcrZ, was identified as the top hit with the highest spectrum counts. AcrA, a periplasmic protein that can bind to AcrB and TolC, forming a tripartite multidrug efflux pump, was also enriched with fewer spectrum counts. The other five enriched proteins, MdtF, ArcB, Trg, MrcA, and LptD, are all membrane proteins. MdtF is a homolog of AcrB. It was shown to function with AcrA to confer bacterial resistance to erythromycin and ciprofloxacin (45). Interestingly, MdtF showed the second-highest spectrum count in the list, although much less than that of AcrB, suggesting its possible interaction with AcrZ. LptD had the lowest spectrum count. It is an outer membrane protein and therefore unlikely to interact directly with AcrZ. ArcB, Trg, and MrcA showed spectrum counts comparable to that of AcrA. Their enrichment in co-IP could be due to indirect interaction *via* AcrB, partially disrupted membrane microdomains, or to differences in their abundance in the different strains (*ΔmgrB*, *ΔsafA*, and *ΔacrZ*).

Co-immunoprecipitated proteins were also eluted with 3xFLAG peptide and analyzed with SDS-PAGE (Fig. 5*C*). AcrB was identified from the top gel band with MS (File S3). However, PhoQ, the known interacting protein of MgrB and SafA, was not identified as one of the enriched proteins with a Z-score above 2 (Fig. S8). Enriched proteins generally had lower spectrum counts than those identified with AcrZ as bait. PhoQ was not identified in the elution by SDS-PAGE either (Fig. 5*C*). We did not observe any bait-specific protein bands in the SafA co-IP samples and no bands in the MgrB co-IP samples on the gel visualized with ReadyBlue protein gel stain, even though all three bait proteins (MgrB, SafA, and AcrZ) were synthesized to similar amounts in the cell-free system based on Western blot results (Fig. S8). Additional higher molecular weight bands were detected in all samples (including the negative control) in the Western blot, which are likely anti-FLAG antibody subunits detached from the magnetic beads and detected by the secondary antibody. As PhoQ activity was modulated by *in vitro*–synthesized MgrB and SafA (Fig. 3*B*), we speculate that the negative co-IP results might be due to the reversible binding of the small proteins to PhoQ and the relatively low abundance of PhoQ sensor kinase in *E. coli*.

To test whether cell-free synthesized MgrB and SafA could bind to their target in a co-IP experimental setup, we performed co-IP with purified recombinant PhoQ. A weak PhoQ band was observed in the bead-only negative control, indicating some nonspecific interactions of PhoQ with the beads. Compared to this negative control, we observed increased amounts of PhoQ in the samples containing FLAG-tagged MgrB and SafA as bait (Fig. 5*D*). Furthermore, a higher amount of PhoQ appeared in the MgrB co-IP samples than in the SafA samples. Considering that both small proteins were synthesized at comparable levels (Fig. S8), these results suggest that MgrB and SafA bind to PhoQ under the co-IP conditions, with MgrB showing stronger interactions. Taken together, these data indicate that our cell-free system is suitable for small protein production and does not interfere with target discovery of small membrane proteins.

## Discussion

In this study, combining lipid sponge droplets with a cell-free system, we have developed an efficient and robust approach to produce functional small membrane proteins in high yields. This allows protein activity assays *in vitro* and assists function discovery of small membrane proteins by identifying their targets. The membrane-rich environment created by lipid sponge droplets provides a large membrane surface area in a three-dimensional space, supporting membrane protein synthesis and simultaneously sequestering the produced protein into the droplets. This unique feature greatly simplifies downstream experimental procedures (*i.e.*, purification and functional assays) and reduces protein loss. Small membrane proteins sensitive to lipid composition could be synthesized in the presence of lipid sponge droplets doped with natural lipids or transferred to liposomes after their synthesis. Encouragingly, both MgrB and SafA were active without separation from lipid sponge droplets, supporting the notion that the components of lipid sponge droplets do not interfere with membrane protein activity.

The success of using the cell-free synthesized AcrZ and identifying the known direct and indirect interactors (AcrB and AcrA) from *E. coli* total membrane highlights the system’s potential in future functional discovery of small membrane proteins. Interestingly, MdtF, another multidrug efflux pump protein, was also robustly enriched *via* co-IP and showed high spectrum counts. MdtF is homologous to AcrB but not constitutively expressed as AcrB. The expression of MdtF was reported to be upregulated in anaerobic growth conditions in *E. coli* (46). Its amount was expected to be less than AcrB in the total membrane isolated from *E. coli* cells grown aerobically in lysogeny broth. Consistently, MS analysis of the solubilized total membrane used for the co-IP experiments indicated that the AcrB amount was about ten times higher than MdtF (data not shown). Despite the lower amount, the enrichment of MdtF using AcrZ as bait strongly suggests that AcrZ interacts and possibly regulates the function of MdtF, though more experimental evidence is needed to support this hypothesis.

Compared to membrane protein synthesis in cell-free systems with liposomes, our system showed a higher yield, producing small membrane proteins in up to micromolar concentrations, which may be due to the unique features of lipid sponge droplets, in particular their internal membrane networks that provide a vast space for membrane protein insertion. In addition to membrane surface area, other contributing factors to *in vitro* transcription-translation may improve the protein yield of our cell-free system, including the codon usage, the ribosomal binding site sequence, the absence of stable secondary structure of mRNA, and the optimal distance between ribosomal-binding site and the start codon (47). We observed an increase in the yield of MgrB, SafA, and sarcolipin when inserting a specific DNA sequence immediately after the start codon and before the FLAG tag, which could be explained by previous reports that the sequence downstream of AUG may interact with 16S ribosomal RNA, assisting translation initiation (43). Along the same lines, to increase the yield of small membrane proteins sensitive to N-terminal tags, one can take advantage of codon redundancy, optimizing the downstream sequence of the start codon to be more complementary to bases 1469 to 1483 of the 16S rRNA (43, 47). At times, we observed additional bands in Western blots, which could be nonspecific binding of the antibodies and/or synthesized protein molecules with higher molecular weight than expected. The latter indicates a possible readthrough of the stop codon, which can be prevented by adding additional stop codons.

The combination of cell-free protein synthesis with lipid sponge droplets simplifies the production of small membrane proteins for biochemical characterization in comparison to *in vivo* approaches and chemical synthesis. Membrane localization of putative small proteins can be rapidly confirmed by microscopy of fluorescent protein fusions in the presence of lipid sponge droplets. Lipid sponge droplets also facilitate further biochemical activity assays and target identification because they enable enrichment of their small membrane protein clients by centrifugation and are directly compatible with co-IP and phosphorylation assays. Similar to other *in vitro* systems, our cell-free approach has limitations: i) the convoluted internal membranous structure in a lipid sponge droplet does not have defined compartmentation; ii) successful target discovery by co-IP appears to depend on stable protein complexes; iii) charged or polar residues within a transmembrane helix may reduce protein yield; and iv) posttranslational modifications are absent. Nevertheless, our system provides a simple protocol for small membrane protein production, functional assays, and target identification. Without cell viability requirements, it enables a high-yield production of toxic small membrane proteins or those that cause cell stress when overexpressed *in vivo*. It is versatile and supports the function of small membrane proteins.

Overall, we present a robust cell-free system as an alternative approach for functional studies of small membrane proteins. With the small reaction volumes and high protein yields, our cell-free system opens up new possibilities for screens that will advance our understanding of small membrane protein biology.

## Experimental procedures

### Chemical reagents and linear DNA templates

N-oleoyl β-D-galactopyranosylamine (GOA) with 99.6% purity was synthesized and analyzed by GLYCON Biochemicals. Octylphenoxypolyethoxyethanol (IGEPAL) was purchased from Sigma (I8896). The C-terminal FLAG-tagged recombinant human programmed cell death 1 ligand 1 protein (PD-L1) was purchased from Biomol (E-PKSH032870.10) and used as a standard for protein quantification. Linear DNA templates were codon-optimized, produced by PCR, and purified using the GeneJET PCR purification kit (Thermo Fisher Scientific). The complete list of linear DNA templates used in this study is summarized in Table S2.

### Cell-free synthesis

The experiments were performed as described previously (35, 36). Briefly, the PURExpress kit (NEB) containing solution A and solution B was used to prepare the transcription-translation reaction. To prepare lipid sponge droplets, a lipid film was prepared by evaporating 42 μl GOA (10 mM) mixed with 100 μl chloroform in a glass vial. The lipid film was rehydrated by vortexing with 21 μl rehydration solution (14 μl PURExpress Solution A, 0.875 μl RNase Inhibitor (NEB), 17 μM IGEPAL (90 mM), 3.465 μl H_2_O) to form lipid sponge droplets. To initiate protein synthesis, 3 μl of the droplet solution was combined with 1.5 μl PURExpress solution B and 0.5 μl DNA template solution to achieve a final concentration of 15 nM. The reactions were carried out at 37 °C at the indicated time. Lipid film and IGEPAL were omitted for cell-free synthesis without lipid sponge droplets.

### Fluorescence microscopy

A 3 μl aliquot of the cell-free reaction mix was pipetted into a lumox dish (Sarstedt) and sealed with a cover glass. The reaction was incubated at 37 °C on stage and observed using a Nikon Eclipse Ti-E inverted fluorescence microscope with a 40× objective. The images and movies were acquired at the indicated time.

### Western blot assay

An equal volume of 2× SDS sample loading buffer was added to a cell-free reaction mix. The mixture was heated at 90 °C for 10 min and loaded onto a 10 to 20% SDS tris-tricine polyacrylamide gel (Thermo Fisher Scientific). Proteins were separated using electrophoresis and transferred to a polyvinylidene fluoride membrane. Total protein was visualized using Revert 700 total protein stain (LI-COR). The polyvinylidene fluoride membrane was then blocked with 5% skim milk in TBST (tris-buffered saline with 0.1% tween 20). Anti-FLAG (Sigma) primary antibody and an IRDye 800CW-conjugated secondary antibody (LI-COR) were used to detect FLAG-tagged proteins. Protein bands were visualized using the Odyssey CLx imaging system (LI-COR) and quantified using ImageJ software.

### Protein yield estimation

The amount of cell-free synthesized small membrane proteins was estimated using Western blot and the commercially available FLAG-tagged protein PD-L1 as a standard. Specifically, cell-free synthesis was stopped at indicated time points by adding the SDS sample loading buffer. The entire reaction mix and 0.1 μg standard protein were then loaded onto an SDS polyacrylamide gel and analyzed with Western blot as described above. The intensity of individual protein bands was calculated by subtracting the background using ImageJ. The amount of cell-free synthesized protein was estimated based on the ratio of protein band intensity and the molarity of the loaded standard protein.

### The overexpression and purification of PhoQ

*E.coli phoQ* with a C-terminal His_6_ tag was cloned into pET Duet-1 vector at NcoI/BamHI restriction sites and transformed into *E.coli* BL21(DE3) strain. The transformed cells were grown in the lysogeny broth medium with ampicillin (100 μg/ml) at 37 °C with vigorous shaking till OD 0.8. Protein expression was then induced with IPTG at a final concentration of 0.5 mM at 16 °C overnight. Cells were harvested, resuspended in the resuspension buffer (50 mM Tris–HCl pH 8, 500 mM NaCl, 10% glycerol, and 0.1 mM PMSF), and lysed using the LM10 microfluidizer at 4 °C. After removing cell debris by centrifugation (11,000*g*) for 10 min, the supernatant was centrifuged again at 100,000*g* for 2 h to pellet the membrane fraction. The pellet was then dissolved in 10 ml resuspension buffer containing 1% LMNG (wt/vol) with gentle shaking at 4 °C for 1 h. The insoluble fraction was removed by centrifugation at 21,000*g* for 45 min. The resulting supernatant was mixed with 5 ml slurry of TALON Superflow resins at 4 °C for 1 h. Subsequently, the TALON Superflow resins were packed into a column and washed with 25 ml washing buffer (50 mM Tris–HCl, pH 8.0, 500 mM NaCl, 10% glycerol, 10 mM imidazole, 0.1 mM PMSF) for three times. PhoQ was eluted with 6 ml elution buffer (50 mM Tris–HCl, pH 8.0, 500 mM NaCl, 10% glycerol, 200 mM imidazole, 0.1 mM PMSF). The washing and elution buffers were not supplemented with additional LMNG due to the high concentration (1%) used in the solubilization step and the low critical micelle concentration of LMNG (0.001%). Purified PhoQ was stored in the final buffer (20 mM Tris–HCl, pH 8.0, 150 mM NaCl).

### Autophosphorylation of PhoQ in the presence of synthesized small proteins

Lipid sponge droplets containing synthesized small proteins were pelleted from a 10 μl cell-free reaction mix by centrifugation at 16,200*g* for 5 min. The supernatant was removed, followed by the addition of purified recombinant PhoQ-His_6_ in the phosphorylation buffer (50 mM Tris–HCl pH 7.5, 200 mM KCl, 0.1 mM EDTA, 10% glycerol, 0.1 mM MgSO4) at the final concentration of 14 μM, resulting in ratios of PhoQ to MgrB and SafA as 24:1 and 12:1, respectively. The autophosphorylation reaction was initiated by adding 0.1 mM ATP containing 10 μCi of [γ-^32^P]ATP and incubated at room temperature for 30 min. The reaction was stopped by adding the SDS sample buffer. The samples were then incubated at 37 °C for 2 min and subsequently loaded onto a 12% precast SDS-polyacrylamide gel (Bio-Rad). Phosphorylated PhoQ was separated from free ATPs by electrophoresis. The gel was exposed to an imaging plate overnight and analyzed with a phosphor imager (Azure Biosystems).

### *In vitro* pull-down assay

The synthesized FLAG-tagged small proteins from a 60 μl cell-free reaction mixture (approximately 100 pmol) were used as bait for the pull-down assay. Total cell membrane was prepared from a 1L culture of *E. coli* strains (*ΔmgrB*, *ΔsafA*, and *ΔacrZ*) and solubilized in the resuspension buffer containing 1% LMNG with the final protein concentration of 8 mg/ml. The synthesized small protein was incubated with 200 μl solubilized crude membrane or purified PhoQ (14 μM) at 4 °C overnight with gentle rotation. After removing possible residual lipid droplets by centrifugation, anti-FLAG M2 magnetic beads (Sigma) were added to the supernatant and incubated at 4 °C for 16 h. The anti-FLAG M2 magnetic beads were washed with 1 ml wash buffer (20 mM Tris–HCl, pH 8.0, 150 mM NaCl, 0.01% LMNG) for ten times and then divided into three equal aliquots: proteins retained on the beads were (1) eluted twice with 100 μl SLS at 90 °C for 10 min followed by MS analysis (details see below); (2) eluted with 3xFLAG peptide (Sigma) at 4 °C followed by SDS-PAGE using a precast Any kD polyacrylamide gel (Bio-Rad); (3) eluted with SDS sample buffer at 95 °C for 10 min followed by SDS-PAGE and Western blot. Proteins in (2) were visualized with Readyblue protein gel stain (Sigma). Protein bands of interest were excised from the gel and subjected to MS analysis.

### Detection of protein interactors using shotgun proteomics

The eluted proteins were reduced using 1 mM TCEP at 90 °C for 10 min. Alkylation of reduced disulfide bonds was performed with 5 mM iodoacetamide at 25 °C in the dark for 30 min. The SP3 approach (48) was used for further sample purification and protein digestion. Briefly, 4 μl SP3 bead slurry from the stock (20 μl beads Sera-mag beads A and B in 100 μl water) was added to the eluate. Then 500 μl acetonitrile was added, and the mixture was incubated at room temperature for 15 min. The beads were separated and washed twice with 70% ethanol, followed by washing with acetonitrile. Trypsin (1 μg) was added to the beads, and protein was digested overnight at 30 °C on a shaking thermomixer. After digestion, the supernatant containing the peptides was collected. Beads were washed with water to increase peptide recovery. The total peptide pool was acidified and purified using Chromabond C18 microspin columns (Macherey-Nagel). In detail, cartridges were prepared by equilibrating with acetonitrile followed by 0.1% TFA. Peptides were loaded onto equilibrated cartridges, washed with buffer containing 5% acetonitrile and 0.1% TFA, and eluted with buffer containing 50% acetonitrile and 0.1% TFA. Solvent was removed, and the dried peptides were redissolved in 0.1% TFA and then analyzed using liquid-chromatography mass spectrometry carried out on an Exploris 480 instrument connected to an Ultimate 3000 RSLC nano and a nanospray flex ion source (all Thermo Fisher Scientific).

Peptide separation was performed on a reverse phase HPLC column (75 μm × 42 cm) packed in-house with C18 resin (2.4 μm; Dr Maisch). The following separating gradient was used: 94% solvent A (0.15% formic acid) and 6% solvent B (99.85% acetonitrile, 0.15% formic acid) to 35% solvent B over 40 min at a flow rate of 300 nl/min. Separated peptides were ionized at a spray voltage of 2.3 kV. The ion transfer tube temperature was set at 275 °C, and 445.12003 m/z was used as internal calibrant. The data acquisition mode was set to obtain one high-resolution MS scan at a resolution of 60,000 full width at half maximum (at m/z 200) followed by MS/MS scans of the most intense ions within 1 s (cycle 1s). To increase the efficiency of MS/MS attempts, the charged state screening modus was enabled to exclude unassigned and singly charged ions. The dynamic exclusion duration was set to 14 s. The ion accumulation time was set to 50 ms (MS) and 50 ms at 17,500 resolution (MS/MS). The automatic gain control was set to 3 x 10^6^ for MS survey scans and 2 x 10^5^ for MS/MS scans. The quadrupole isolation was 1.5 m/z, and the collision was induced with an higher-energy collision dissociation collision energy of 27%.

MS raw data was then searched using Sequest HT *via* the Proteome Discoverer platform (Thermo Fisher Scientific) against an *E.coli* UniProt database. The search criteria were set as follows: full tryptic specificity was required (cleavage after lysine or arginine residues); three missed cleavages were allowed; carbamidomethylation (C) was set as fixed modification; oxidation (M), deamidation (N, Q) as variable modification. Mass tolerance was set to 10 ppm on precursors and 0.02 Da on fragments. The results were then imported into Scaffold (v5, Proteome Software). Within Scaffold, the data was obtained with protein false discovery rate set to 1%, and total spectrum counts were exported for further analysis. As described before (49), we performed Z-transformation of log ratios of the spectrum counts found in AcrZ-IPs *versus* unrelated bait protein IP-MS experiments (MgrB-IP and SafA-IP). For calculations of spectrum log ratios, “0” was replaced with “0.5” as a background value. Proteins were considered enriched when a minimum Z-score of “2” was reached in two independently performed experiments.

## Data availability

The Proteomics MS raw data is available *via* ProteomeXchange with the identifier PXD051183. Username: reviewer_pxd051183@ebi.ac.uk, Password: KtWfrl3w.

## Supporting information

This article contains supporting information.

## Conflict of interest

The authors declare that they have no conflicts of interest with the contents of this article.
