# Supplementary material for: A cell-free system for functional studies of small membrane proteins
Source: J Biol Chem. 2024 Oct 1;300(11):107850. doi: 10.1016/j.jbc.2024.107850 (PMC11539335; doi:10.1016/j.jbc.2024.107850)
Supplement: Supporting information [file mmc1.docx]

**SUPPORTING INFORMATION**

**Title:** A cell-free system for functional studies of small membrane proteins

**Authors:** Shan Jiang ^1^, Gülce Çelen^1^, Timo Glatter^1^, Henrike Niederholtmeyer ^1, 2*^, Jing Yuan ^1*^

1 Max Planck Institute for Terrestrial Microbiology and Center for Synthetic Microbiology, 35043 Marburg, Germany.

2 Technical University of Munich, Campus Straubing for Biotechnology and Sustainability, 94315 Straubing, Germany.

* Corresponding author

**Supporting Information List:**

**Table S1:** The natural properties of small membrane proteins.

**Table S2:** The sequences of linear DNA templates used in this study.

**Fig. S1:** Quantification of the Western blot bands in Fig. 2B.

**Fig. S2:** Quantification of the Western blot bands in Fig. 2C.

**Fig. S3:** Functional assay of tag-free small membrane proteins.

**Fig. S4:** Quantification of the Western blot bands in Fig. 4A.

**Fig. S5:** Quantification of the Western blot bands in Fig. 4B.

**Fig. S6:** Cell-free synthesis of sarcolipin and its N to L mutant.

**Fig. S7:** Quantification of the Western blot bands in Fig 4C.

**Fig. S8:** Identification of interacting targets of MgrB and SafA.

**TableS1.** The natural properties of small membrane proteins.

|  | MgrB  (47 aa) | SafA  (65 aa) | AcrZ  (49 aa) | Sarcolipin  (31 aa) |
| --- | --- | --- | --- | --- |
| **Sequences** | MKKFRWVVLVVVVLACLLLWAQVFNMMCDQDVQFFSGICAINQFIPW | MHATTVKNKITQRDNYKEIMSAIVVVLLLTLTLIAIFSAIDQLSISEMGRIARDLTHFIINSLQG | MLELLKSLVFAVIMVPVVMAIILGLIYGLGEVFNIFSGVGKKDQPGQNH | MGINTRELFLNFTIVLITVILMWLLVRSYQY |
| **AlphaFold 2**  **model** | 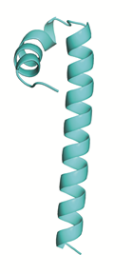 | 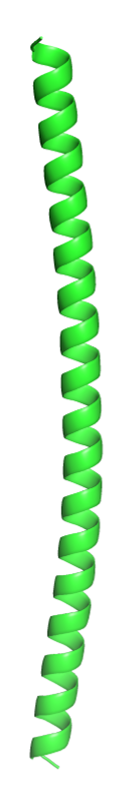 | 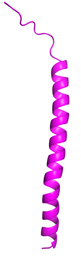 | 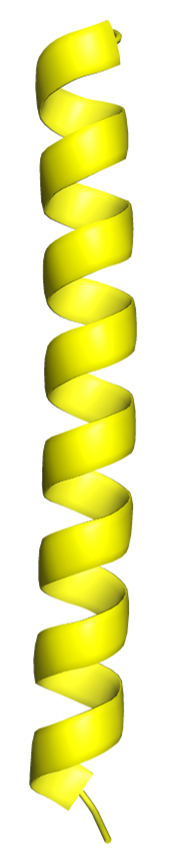 |
| **Hydrophobic ratio** | 0.57 | 0.46 | 0.51 | 0.58 |
| **Positive charge ratio** | 0.06 | 0.12 | 0.08 | 0.06 |
| **Negative charge ratio** | 0.04 | 0.08 | 0.06 | 0.03 |
| **Isoelectric point** | 7.331 | 8.137 | 6.937 | 8.347 |
| **Transmembrane helix** | WVVLVVVVLACLLLWAQVF | IMSAIVVVLL  LTLTLIAIFSA | LVFAVIMVP  VVMAIILGLI  YG | LFLNFTIVLI  TVILMWLLV |

**Table S2.** The sequences of linear DNA templates were used in this study.

| **Linear DNA templates** | **Sequences**  **(T7 promoter, ribosomal binding site, affinity tag, ORF, T7 terminator)** |
| --- | --- |
| P_T7_-*mNeonGreen-mgrB* | GCGAATTAATACGACTCACTATAGGCGGATAACAATTTCACACAGGAAACAGACCATGGTTTCTAAGGGTGAAGAAGACAACATGGCTTCTTTGCCAGCTACTCACGAATTGCACATCTTCGGTTCTATCAACGGTGTTGACTTCGACATGGTTGGTCAAGGTACTGGTAACCCAAACGACGGTTACGAAGAATTGAACTTGAAGTCTACTAAGGGTGACTTGCAATTCTCTCCATGGATCTTGGTTCCACACATCGGTTACGGTTTCCACCAATACTTGCCATACCCAGACGGTATGTCTCCATTCCAAGCTGCTATGGTTGACGGTTCTGGTTACCAAGTTCACAGAACTATGCAATTCGAAGACGGTGCTTCTTTGACTGTTAACTACAGATACACTTACGAAGGTTCTCACATCAAGGGTGAAGCTCAAGTTAAGGGTACTGGTTTCCCAGCTGACGGTCCAGTTATGACTAACTCTTTGACTGCTGCTGACTGGTGTAGATCTAAGAAGACTTACCCAAACGACAAGACTATCATCTCTACTTTCAAGTGGTCTTACACTACTGGTAACGGTAAGAGATACAGATCTACTGCTAGAACTACTTACACTTTCGCTAAGCCAATGGCTGCTAACTACTTGAAGAACCAACCAATGTACGTTTTCAGAAAGACTGAATTGAAGCACTCTAAGACTGAATTGAACTTCAAGGAATGGCAAAAGGCTTTCACTGACGTTATGGGTATGGACGAATTGTACAAGggttcttccggctcatcaggctctagtATGAAAAAGTTTCGATGGGTCGTTCTGGTTGTCGTGGTGTTGGCTTGCTTGCTGCTTTGGGCGCAGGTATTCAACATGATGTGCGATCAGGATGTACAATTTTTCAGCGGAATTTGTGCCATTAACCAGTTTATCCCGTGGTGACTAGCATAACCCCTTGGGGCCTCTAAACGGGTCTTGAGGGGTTTTTTG |
| P_T7_-*mNeonGreen-safA* | GCGAATTAATACGACTCACTATAGGCGAGCTCGGTACCACAACTTAAGGAGGTATTCATGGTTTCTAAGGGTGAAGAAGACAACATGGCTTCTTTGCCAGCTACTCACGAATTGCACATCTTCGGTTCTATCAACGGTGTTGACTTCGACATGGTTGGTCAAGGTACTGGTAACCCAAACGACGGTTACGAAGAATTGAACTTGAAGTCTACTAAGGGTGACTTGCAATTCTCTCCATGGATCTTGGTTCCACACATCGGTTACGGTTTCCACCAATACTTGCCATACCCAGACGGTATGTCTCCATTCCAAGCTGCTATGGTTGACGGTTCTGGTTACCAAGTTCACAGAACTATGCAATTCGAAGACGGTGCTTCTTTGACTGTTAACTACAGATACACTTACGAAGGTTCTCACATCAAGGGTGAAGCTCAAGTTAAGGGTACTGGTTTCCCAGCTGACGGTCCAGTTATGACTAACTCTTTGACTGCTGCTGACTGGTGTAGATCTAAGAAGACTTACCCAAACGACAAGACTATCATCTCTACTTTCAAGTGGTCTTACACTACTGGTAACGGTAAGAGATACAGATCTACTGCTAGAACTACTTACACTTTCGCTAAGCCAATGGCTGCTAACTACTTGAAGAACCAACCAATGTACGTTTTCAGAAAGACTGAATTGAAGCACTCTAAGACTGAATTGAACTTCAAGGAATGGCAAAAGGCTTTCACTGACGTTATGGGTATGGACGAATTGTACAAGaagcttggctgttttggcggaggatccATGCATGCGACCACAGTGAAAAACAAAATCACGCAAAGAGACAACTATAAAGAAATCATGTCTGCAATTGTGGTTGTCTTATTACTGACACTTACGTTGATAGCCATTTTTTCGGCAATTGATCAGCTGAGTATTTCAGAAATGGGTCGCATTGCAAGAGATCTTACACATTTCATTATCAATAGTTTGCAAGGCTGACTAGCATAACCCCTTGGGGCCTCTAAACGGGTCTTGAGGGGTTTTTTG |
| P_T7_-*mNeonGreen* | GTCTTCACCTCGAGGATCTTAAGGCTAGAGTAATACGACTCACTATAGGGAGATGTGGTCTAGACATTCCAGGTTAAGAAGGAGGAAAAAAAAATGGTTTCTAAGGGTGAAGAAGACAACATGGCTTCTTTGCCAGCTACTCACGAATTGCACATCTTCGGTTCTATCAACGGTGTTGACTTCGACATGGTTGGTCAAGGTACTGGTAACCCAAACGACGGTTACGAAGAATTGAACTTGAAGTCTACTAAGGGTGACTTGCAATTCTCTCCATGGATCTTGGTTCCACACATCGGTTACGGTTTCCACCAATACTTGCCATACCCAGACGGTATGTCTCCATTCCAAGCTGCTATGGTTGACGGTTCTGGTTACCAAGTTCACAGAACTATGCAATTCGAAGACGGTGCTTCTTTGACTGTTAACTACAGATACACTTACGAAGGTTCTCACATCAAGGGTGAAGCTCAAGTTAAGGGTACTGGTTTCCCAGCTGACGGTCCAGTTATGACTAACTCTTTGACTGCTGCTGACTGGTGTAGATCTAAGAAGACTTACCCAAACGACAAGACTATCATCTCTACTTTCAAGTGGTCTTACACTACTGGTAACGGTAAGAGATACAGATCTACTGCTAGAACTACTTACACTTTCGCTAAGCCAATGGCTGCTAACTACTTGAAGAACCAACCAATGTACGTTTTCAGAAAGACTGAATTGAAGCACTCTAAGACTGAATTGAACTTCAAGGAATGGCAAAAGGCTTTCACTGACGTTATGGGTATGGACGAATTGTACAAGTAACGACTCAGGCTGCTACTCAAAACTAGCATAACCCCTTGGGGCCTCTAAACGGGTCTTGAGGGGTTTTTTG |
| P_T7_-*flag-mgrB* | GCGAATTAATACGACTCACTATAGGAATTGTGAGCGGATAACAATTTCACACAGGAAACAGACCATGGATTATAAAGATGATGATGATAAAggtggcggaggatccATGAAAAAGTTTCGATGGGTCGTTCTGGTTGTCGTGGTGTTGGCTTGCTTGCTGCTTTGGGCGCAGGTATTCAACATGATGTGCGATCAGGATGTACAATTTTTCAGCGGAATTTGTGCCATTAACCAGTTTATCCCGTGGTGACTAGCATAACCCCTTGGGGCCTCTAAACGGGTCTTGAGGGGTTTTTTG |
| P_T7_- *flag-safA* | GCGAATTAATACGACTCACTATAGGTCTCCATACCCGTTTTTTTGGGCTAGCGAGGAGTTCGAGCTCATGGATTATAAAGATGATGATGATAAAggtggcggtggcagcATGCATGCGACCACAGTGAAAAACAAAATCACGCAAAGAGACAACTATAAAGAAATCATGTCTGCAATTGTGGTTGTCTTATTACTGACACTTACGTTGATAGCCATTTTTTCGGCAATTGATCAGCTGAGTATTTCAGAAATGGGTCGCATTGCAAGAGATCTTACACATTTCATTATCAATAGTTTGCAAGGCTGACTAGCATAACCCCTTGGGGCCTCTAAACGGGTCTTGAGGGGTTTTTTG |
| P_T7_-*mgrB* | GCGAATTAATACGACTCACTATAGGTTTTTTTGGGCTAGCGAGGAGTTCGAGCTCATGAAAAAGTTTCGATGGGTCGTTCTGGTTGTCGTGGTGTTGGCTTGCTTGCTGCTTTGGGCGCAGGTATTCAACATGATGTGCGATCAGGATGTACAATTTTTCAGCGGAATTTGTGCCATTAACCAGTTTATCCCGTGGTGACTAGCATAACCCCTTGGGGCCTCTAAACGGGTCTTGAGGGGTTTTTTG |
| P_T7_- *safA* | GCGAATTAATACGACTCACTATAGGCGGATAACAATTTCACACAGGAAACAGACCATGCATGCGACCACAGTGAAAAACAAAATCACGCAAAGAGACAACTATAAAGAAATCATGTCTGCAATTGTGGTTGTCTTATTACTGACACTTACGTTGATAGCCATTTTTTCGGCAATTGATCAGCTGAGTATTTCAGAAATGGGTCGCATTGCAAGAGATCTTACACATTTCATTATCAATAGTTTGCAAGGCTGACTAGCATAACCCCTTGGGGCCTCTAAACGGGTCTTGAGGGGTTTTTTG |
| P_T7_-*acrZ-flag* | GAAATTAATACGACTCACTATAGGGGAATTGTGAGCGGATAACAATTCCCCTGTAGAAATAATTTTGTTTAACTTTAATAAGGAGATATACCATGGGCTTAGAGTTATTAAAAAGTCTGGTATTCGCCGTAATCATGGTACCTGTCGTGATGGCCATCATCCTGGGTCTGATTTACGGTCTTGGTGAAGTATTCAACATCTTTTCTGGTGTTGGTAAAAAAGACCAGCCCGGACAAAATCATggcggtggcggtagcGATTATAAAGATGATGATGATAAATGACTAGCATAACCCCTTGGGGCCTCTAAACGGGTCTTGAGGGGTTTTTTG |
| P_T7_- *flag-sacrcolipin* | GCGAATTAATACGACTCACTATAGGAATTGTGAGCGGATAACAATTTCACACAGGAAACAGACCATGGATTATAAAGATGATGATGATAAAggtggcggaggatccATGGGCATTAACACCCGTGAGCTGTTTCTGAACTTCACTATTGTCTTGATTACGGTTATTCTTATGTGGCTCCTTGTGCGTTCCTATCAGTACTGACTAGCATAACCCCTTGGGGCCTCTAAACGGGTCTTGAGGGGTTTTTTG |
| P_T7_- *flag-sacrcolipin*(N11L) | GCGAATTAATACGACTCACTATAGGAATTGTGAGCGGATAACAATTTCACACAGGAAACAGACCATGGATTATAAAGATGATGATGATAAAggtggcggaggatccATGGGCATTAACACCCGTGAGCTGTTTCTGCTGTTCACTATTGTCTTGATTACGGTTATTCTTATGTGGCTCCTTGTGCGTTCCTATCAGTACTGACTAGCATAACCCCTTGGGGCCTCTAAACGGGTCTTGAGGGGTTTTTTG |
| P_T7_-5aa *-flag-safA* | GAAATTAATACGACTCACTATAGGGGAATTGTGAGCGGATAACAATTCCCCTGTAGAAATAATTTTGTTTAACTTTAATAAGGAGATATACCATGGGCTTAGAGTTAGATTATAAAGATGATGATGATAAAggtggcggtggcagcATGCATGCGACCACAGTGAAAAACAAAATCACGCAAAGAGACAACTATAAAGAAATCATGTCTGCAATTGTGGTTGTCTTATTACTGACACTTACGTTGATAGCCATTTTTTCGGCAATTGATCAGCTGAGTATTTCAGAAATGGGTCGCATTGCAAGAGATCTTACACATTTCATTATCAATAGTTTGCAAGGCTGACTAGCATAACCCCTTGGGGCCTCTAAACGGGTCTTGAGGGGTTTTTTG |
| P_T7_-5aa*-flag-sarcolipin* | GAAATTAATACGACTCACTATAGGGGAATTGTGAGCGGATAACAATTCCCCTGTAGAAATAATTTTGTTTAACTTTAATAAGGAGATATACCATGGGCTTAGAGTTAGATTATAAAGATGATGATGATAAAggtggcggaggatccATGGGCATTAACACCCGTGAGCTGTTTCTGAACTTCACTATTGTCTTGATTACGGTTATTCTTATGTGGCTCCTTGTGCGTTCCTATCAGTACTGACTAGCATAACCCCTTGGGGCCTCTAAACGGGTCTTGAGGGGTTTTTTG |
| P_T7_-5aa*-flag-mgrB* | GAAATTAATACGACTCACTATAGGGGAATTGTGAGCGGATAACAATTCCCCTGTAGAAATAATTTTGTTTAACTTTAATAAGGAGATATACCATGGGCTTAGAGTTAGATTATAAAGATGATGATGATAAAggtggcggaggatccATGAAAAAGTTTCGATGGGTCGTTCTGGTTGTCGTGGTGTTGGCTTGCTTGCTGCTTTGGGCGCAGGTATTCAACATGATGTGCGATCAGGATGTACAATTTTTCAGCGGAATTTGTGCCATTAACCAGTTTATCCCGTGGTGACTAGCATAACCCCTTGGGGCCTCTAAACGGGTCTTGAGGGGTTTTTTG |


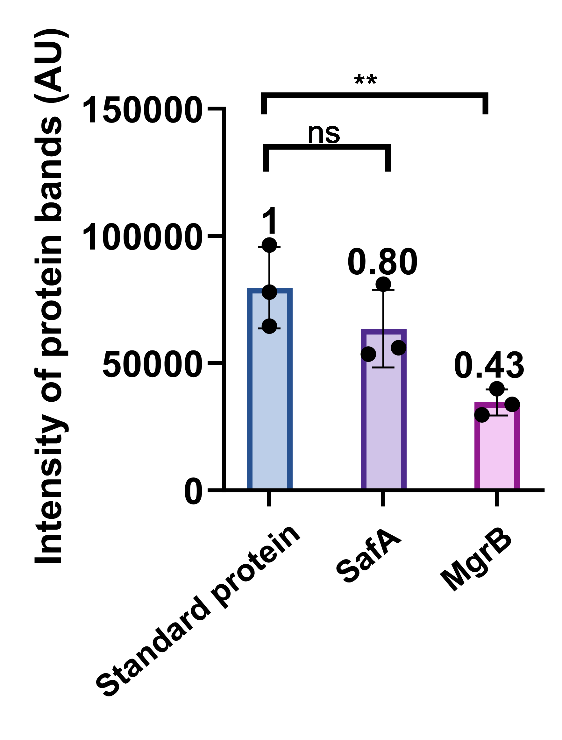


**Fig. S1. Quantification of the Western blot bands in Fig. 2B.** Compared to 0.1 µg FLAG-tagged standard protein (3.8 pmol), the amount of synthesized SafA in the presence of lipid sponge droplets in a 5µl reaction was 608 nM. The amount of synthesized MgrB reached 327 nM. The number above each bar represents the fold change of protein band intensity compared to the standard protein. ImageJ was used for quantification. The error bars represent standard deviations from three independent experiments. The p-value is calculated using GraphPad Prism 10.


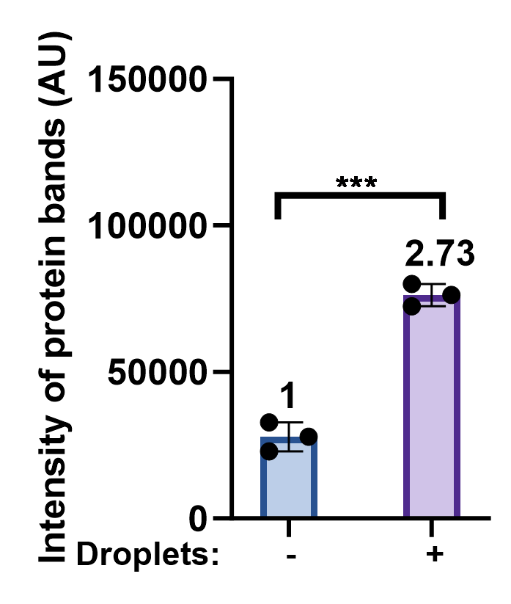


**Fig. S2. Quantification of the Western blot bands in Fig. 2C**. Compared to synthesizing SafA without droplets, the amount of synthesized SafA in the presence of lipid sponge droplets showed a 2.73-fold increase. The error bars with the raw data points represent standard deviations from two independent experiments. The p-value is calculated using GraphPad Prism 10.


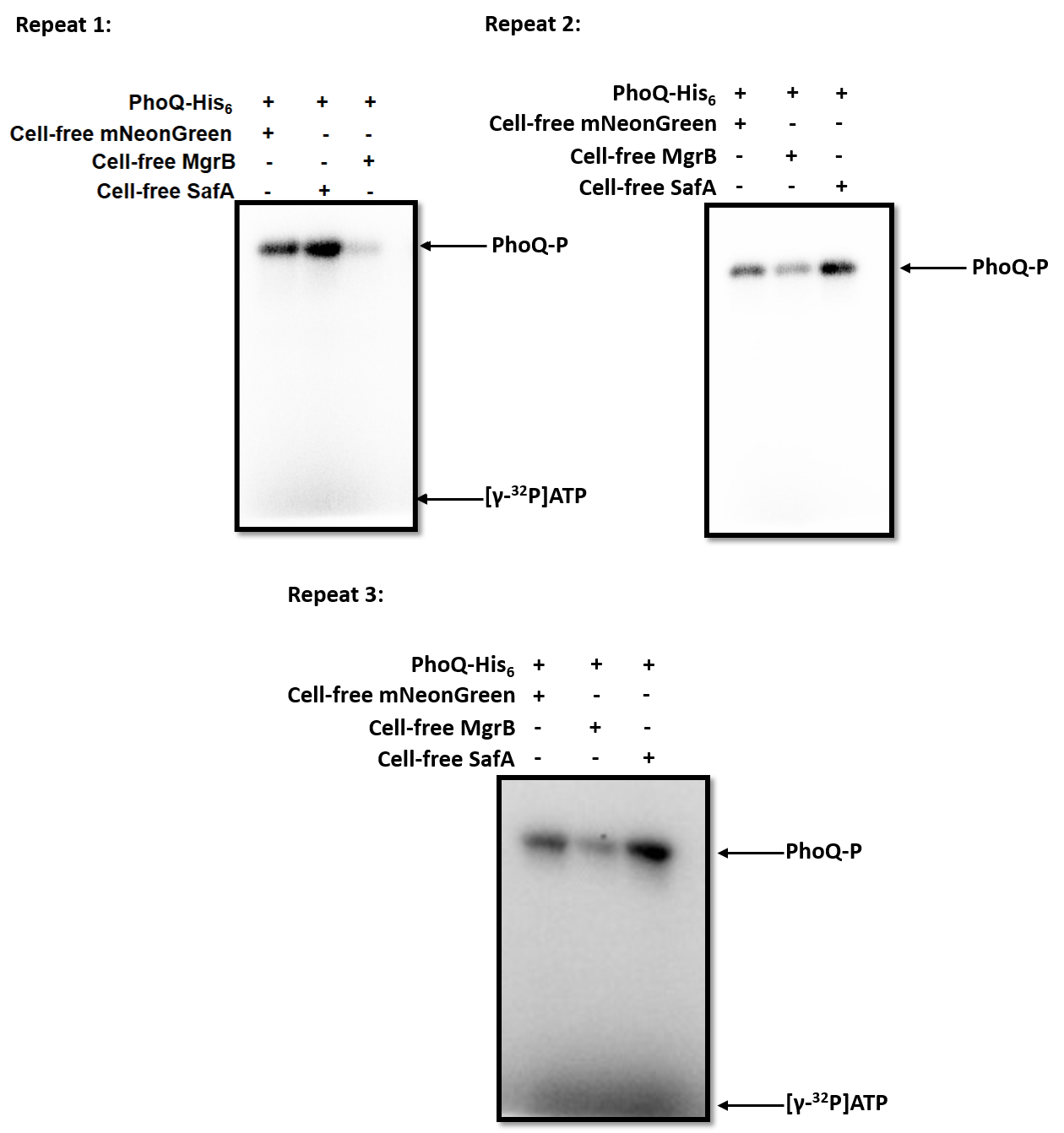


**Fig. S3. Functional assay of tag-free small membrane proteins**. The autophosphorylation reactions were performed as in Fig. 3B. Phosphorylated PhoQ in the presence of synthesized proteins were analyzed with SDS-PAGE and detected by phosphorimager.

**
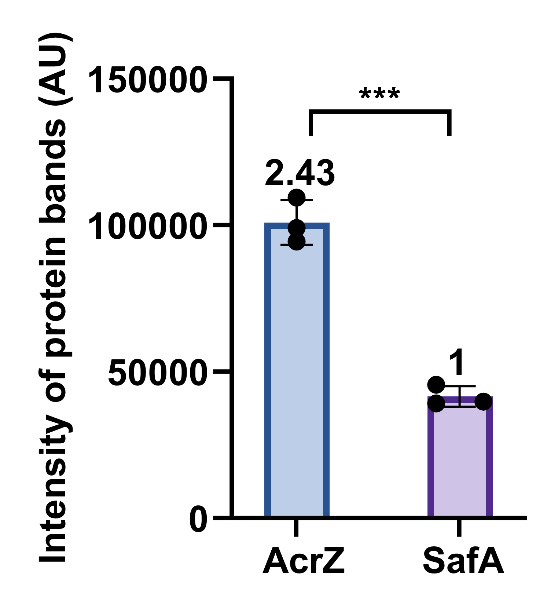
**

**Fig. S4. Quantification of the Western blot bands in Fig. 4A**. Compared to SafA, the amount of synthesized AcrZ showed a more than 2-fold increase (about 1.4 µM). The error bars represent standard deviations from three independent experiments. The p-value is calculated using GraphPad Prism 10.

**
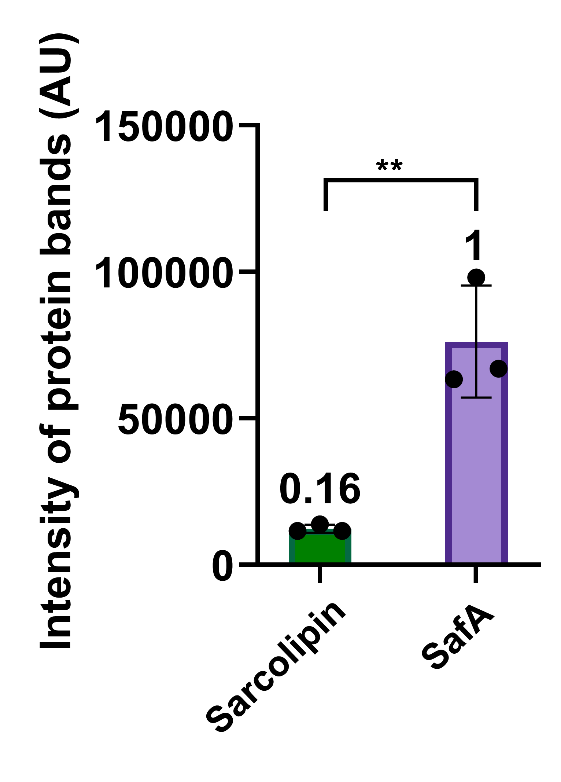
**

**Fig. S5.** **Quantification of the Western blot bands in Fig. 4B**. Compared to SafA, the amount of synthesized sarcolipin showed a 0.16-fold decrease (about 90 nM). The error bars represent standard deviations from three independent experiments. The p-value is calculated using GraphPad Prism 10.


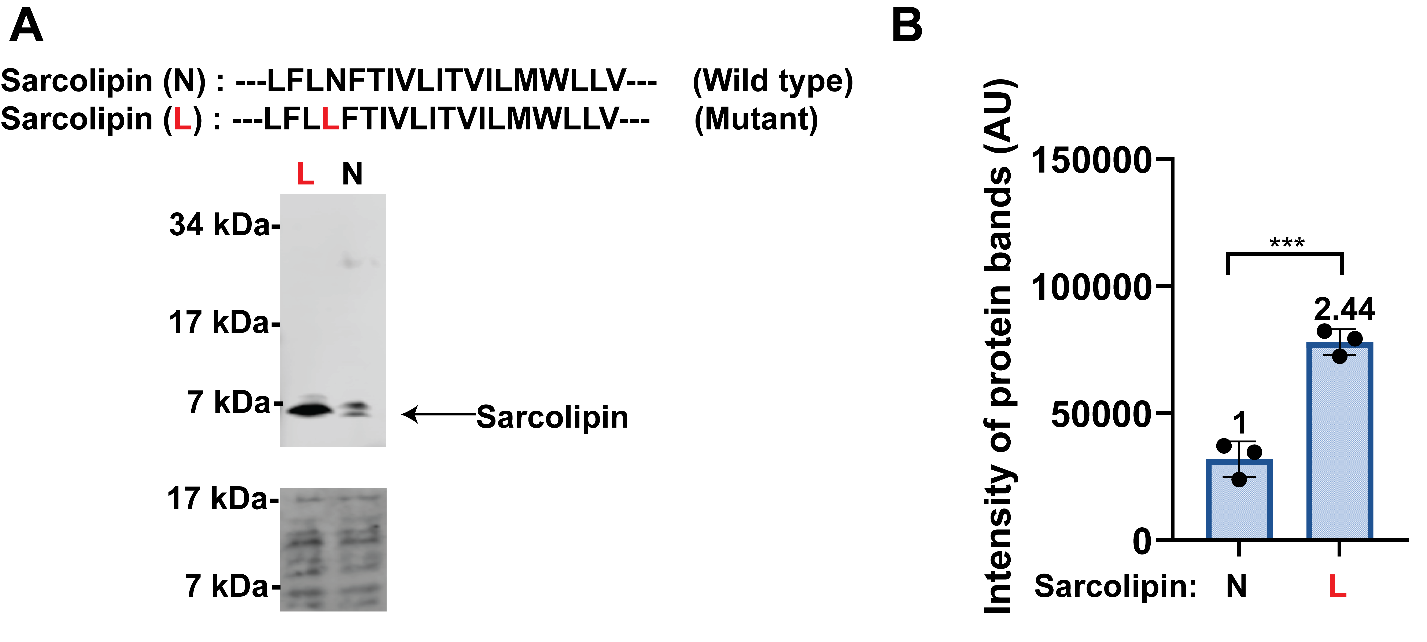


**Fig.S6. Cell-free synthesis of sarcolipin and its N to L mutant.** (A) Western blot analysis of synthesized sarcolipin and its mutant in the presence of lipid sponge droplets. The total protein stain of the PVDF membranes serves as loading control. (B) Quantification of the Western blot bands in A using ImageJ software. Compared to wild-type sarcolipin, the amount of the synthesized mutant sarcolipin showed a 2.44-fold increase. The error bars represent standard deviations from three independent experiments. The p-value is calculated using GraphPad Prism 10.


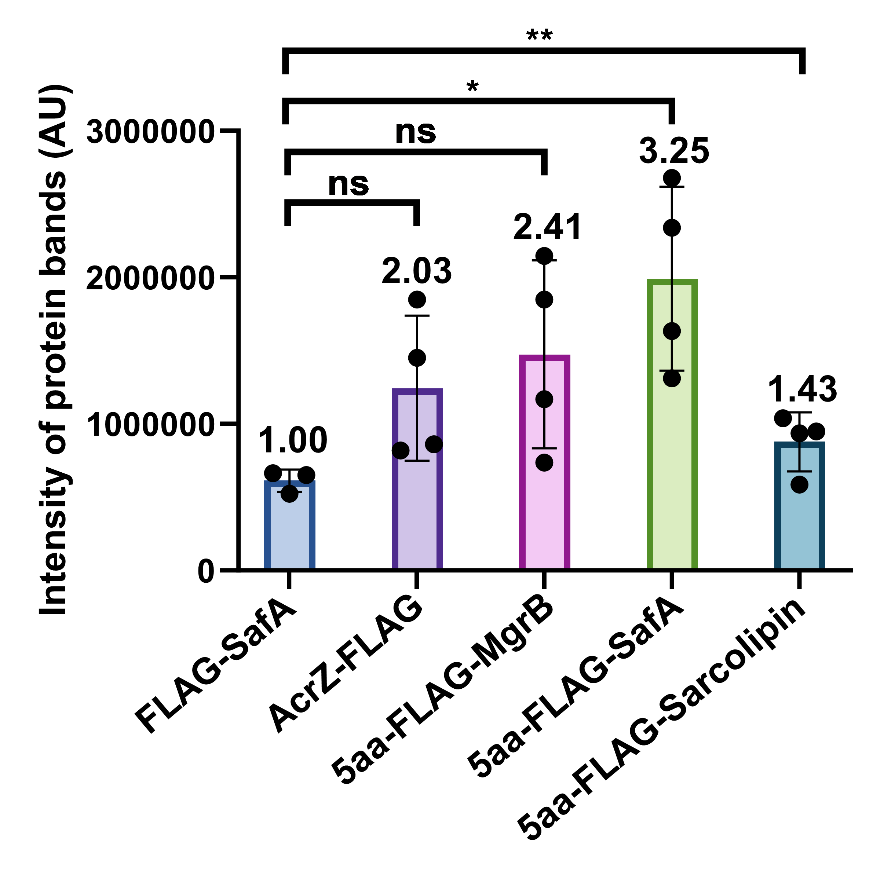


**Fig.S7.** **Quantification of the Western blot bands in Fig 4C.** The number above each bar represents the fold change when compared to the intensity of the AcrZ-FLAG band in the Western blot. Based on the results in Fig. 2B and Fig. S1, about 600 nM FLAG-SafA was produced in a 5μl reaction. The addition of 5aa increased the yield of MgrB, SafA and sarcolipin at or near the micromolar range (1446 nM, 1950 nM, and 858 nM respectively). The error bars represent standard deviations from at least three independent experiments. The p-value is calculated using GraphPad Prism 10.


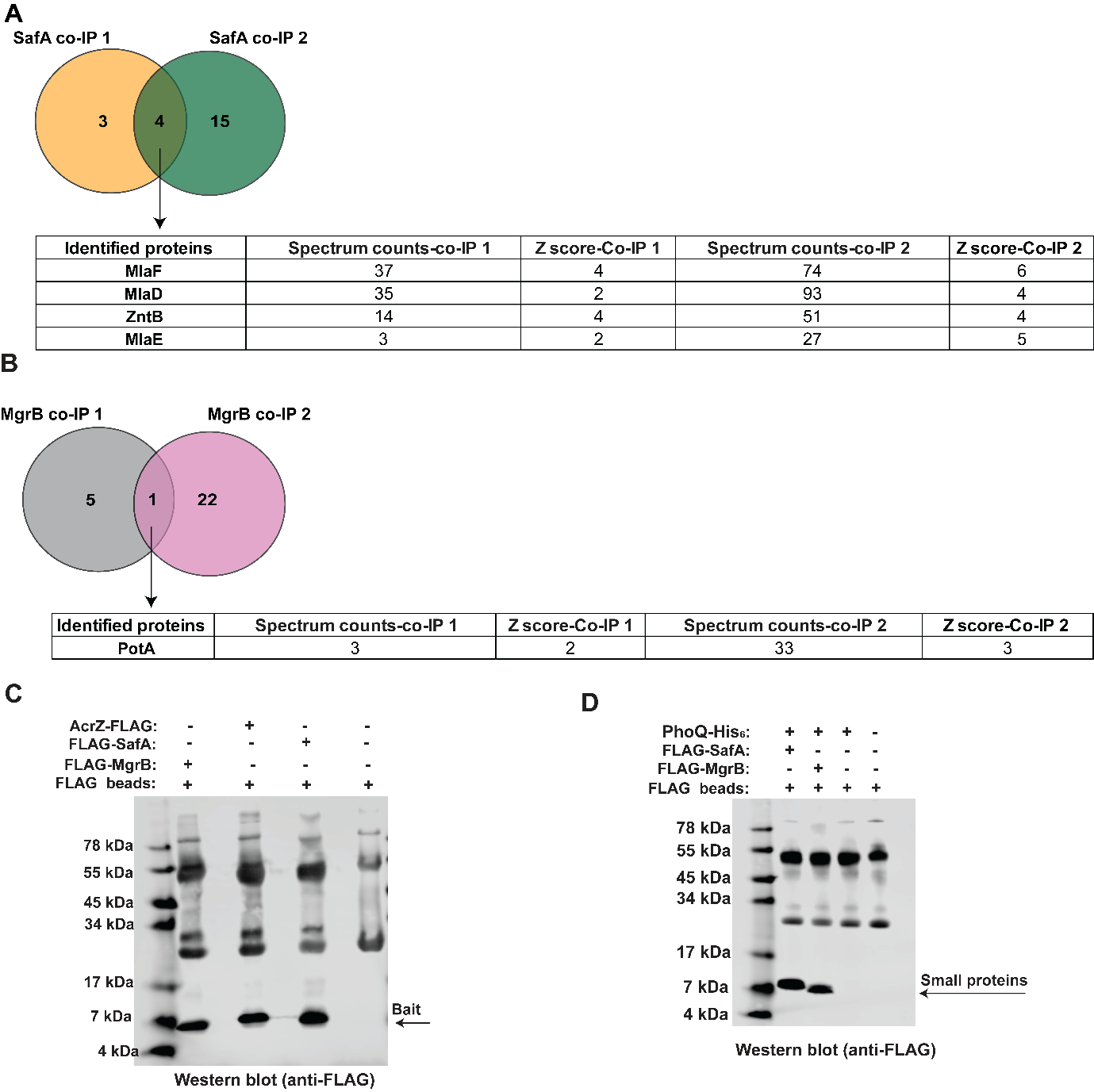


**Fig.S8.** **Identification of interacting targets of MgrB and SafA.** Using synthesized FLAG-tagged SafA (A) and MgrB (B) as bait, co-immunoprecipitated proteins were identified via MS. Proteins with a minimum Z-score of 2 in two independent experiments are shown and considered enriched. The tables list enriched proteins with spectrum counts. (C) Western blot verification of synthesized FLAG-tagged MgrB, AcrZ, and SafA in the co-IP experiment in Figure 5C. (D) Western blot verification of the synthesized FLAG-MgrB and FLAG-SafA in the co-IP experiment in Figure 5D.

**Movie S1.** Cell-free synthesis of mNeonGreen-MgrB in the presence of lipid sponge droplets shown as a time-lapse movie of merged transmitted light and GFP fluorescence images. The movie is associated with the images in Figure 1A.
